# Do Photobiont Switch and Cephalodia Emancipation Act as Evolutionary Drivers in the Lichen Symbiosis? A Case Study in the Pannariaceae (Peltigerales)

**DOI:** 10.1371/journal.pone.0089876

**Published:** 2014-02-24

**Authors:** Nicolas Magain, Emmanuël Sérusiaux

**Affiliations:** Evolution and Conservation Biology Unit, University of Liège, Liège, Belgium; University of California-Riverside, United States of America

## Abstract

Lichen symbioses in the Pannariaceae associate an ascomycete and either cyanobacteria alone (usually *Nostoc*; bipartite thalli) or green algae and cyanobacteria (cyanobacteria being located in dedicated structures called cephalodia; tripartite thalli) as photosynthetic partners (photobionts). In bipartite thalli, cyanobacteria can either be restricted to a well-delimited layer within the thallus (‘pannarioid’ thalli) or spread over the thallus that becomes gelatinous when wet (‘collematoid’ thalli). We studied the collematoid genera *Kroswia* and *Physma* and an undescribed tripartite species along with representatives of the pannarioid genera *Fuscopannaria*, *Pannaria* and *Parmeliella*. Molecular inferences from 4 loci for the fungus and 1 locus for the photobiont and statistical analyses within a phylogenetic framework support the following: (a) several switches from pannarioid to collematoid thalli occured and are correlated with photobiont switches; the collematoid genus *Kroswia* is nested within the pannarioid genus *Fuscopannaria* and the collematoid genus *Physma* is sister to the pannarioid *Parmeliella mariana* group; (b) *Nostoc* associated with collematoid thalli in the Pannariaceae are related to that of the Collemataceae (which contains only collematoid thalli), and never associated with pannarioid thalli; *Nostoc* associated with pannarioid thalli also associate in other families with similar morphology; (c) ancestors of several lineages in the Pannariaceae developed tripartite thalli, bipartite thalli probably resulting from cephalodia emancipation from tripartite thalli which eventually evolved and diverged, as suggested by the same *Nostoc* present in the collematoid genus *Physma* and in the cephalodia of a closely related tripartite species; Photobiont switches and cephalodia emancipation followed by divergence are thus suspected to act as evolutionary drivers in the family Pannariaceae.

## Introduction

Several spectacular aspects of the lichen symbiosis have come to light recently, the most surprizing for the general public and the most promising for evolutionary studies being the multiple variations of the association between the mycobiont and photobiont partners. The lichen as the icon of consensual and stable symbiosis between two very different partners “for better and for worse” is not the model that molecular studies have produced in recent years. Indeed, some mycobionts can incorporate several algal genotypes in their thallus [1]–[3], or even different algal species [4]–[5]. Several phylogenetic studies have demonstrated that photobiont switching is rather widespread [6], even in obligatory sterile taxa where both partners are dispersed together, and may occur repeatedly over evolutionary timescales [7]. Studies of the genetic diversity of both partners within a geographical context revealed that mycobionts can recruit several lineages of photobionts, allowing for ecotypic differentiation and thus for colonization of different ecological niches and distribution [6], [8]. Those multiple variations in the association between the partners involved in the lichen symbiosis may take part in their evolutionary trajectory and we here address that matter for a lichen family (the Pannariaceae) in which several very different types of thalli occur together with variation in the number of photobionts involved in their construction.

The Peltigerales, a strongly supported lineage within the Lecanoromycetes, contains many well-known lichen genera, such as *Lobaria, Peltigera* and *Sticta*, within 10 families [9]–[12], including the Collemataceae and the Pannariaceae, two families that will be mentioned in this paper.

Within the Peltigerales, symbiosis includes two different lineages of photobionts [10]: (a) cyanobacteria mostly belonging to the genus *Nostoc*, or to *Scytonema*, *Hyphomorpha* and other taxa in the Scytonemataceae and Rivulariaceae; (b) green algae, mainly assigned to the genera *Coccomyxa, Dictyochloropsis, Myrmecia*, all belonging to the Trebouxiophyceae. The number of photobionts associated with the mycobiont provides the ground for the distinction of bi- and tripartite lichens, the latter case being much more diverse in the way of allocating space for the cyanobacteria [13]–[15]:

- association with a single photobiont partner, either a cyanobacteria or a green algae; these thalli are bipartite and are referable to the cyanolichens or the chlorolichens, respectively [16];
- association with two partners, a cyanobacteria and a green algae and corresponding thalli referred to as tripartite thalli [17]; the topological organization of the partners can vary : (b1) both photobionts can be present in a dedicated layer within the thallus (chloro-cyanolichen; see [16]); (b2) the green photobiont is present in a dedicated layer within the thallus whilst cyanobacteria are confined to dedicated and morphologically recognizable organs, named cephalodia [18]; (b3) production of two different thallus types, either living independently from one another or being closely associated, one with the cyanobacteria and the other one with the green algae; these structures are referred to as « photosymbiodemes », « photopairs » or « photomorphs » and can be morphologically rather similar or very much different one from the other – in the latter case the cyanomorph has a *Dendriscocaulon*-like morphology [14].

Further two different types of cyanobacterial bipartite thallus can be distinguished on the basis of their response to changes in water availability [19]. A first type is characterized by thalli that swell considerably and become very much gelatinous when wet, and return to a rather brittle and crumpled condition when dry, while the second type has thalli that do not radically change when water availability varies, albeit strong changes in color can occur. The first type is associated with a homoiomerous thallus anatomy, that is absence of a specialized photobiont layer, with chains of *Nostoc* with thick mucilaginous walls being easily recognized and present throughout the thallus thickness, an upper cortex being absent or present; it will be hereafter referred to as the collematoid thallus type. The second type of thallus is heteromerous, that is with a usually very distinct photobiont layer present under the upper cortex (which is always present) and *Nostoc* (or other genera) or green algal cells compacted and assembled in clusters. Within the second group, several morphotypes can be distinguished, ranging from nearly crustose to large foliose and dendroid-fruticose; the pannarioid type refers to a squamulose to foliose thallus developed over a black prothallus. Within the Peltigerales, a thallus associated with cyanobacteria can either belong to the collematoid or to other types, incl. the pannarioid type; on the other hand, thalli associated with green algae never belong to the collematoid type.

The assignment of collematoid taxa to a single family (Collemataceae) has been the rule for a long time [20]–[25]. Several exceptions are worth mentioning as they anticipate the more recent resolution of several genera outside the family: the collematoid genera *Kroswia* and *Lepidocollema* and the species *Pannaria santessonii* have been assigned to the Pannariaceae [26]–[30] while the genus *Hydrothyria* was recognized as close to *Peltigera* [26], [31].

Access to molecular data and their optimization with modern statistical methods caused many relocation of collematoid taxa: to the genus *Peltigera* for both species of *Hydrothyria* [32]–[33]; to another family within the Peltigerales, the Massalongiaceae for the genera *Leptochidium* and *Massalongia* [11]; to the Pannariaceae for several genera (*Leciophysma, Leptogidium, Physma, Ramalodium*, *Staurolemma, Steineropsis*) and a species of *Santessoniella* (*S. saximontana*) [19], [34], [35], [36]; and to an unrelated family, the Arctomiaceae [37] for *Collema fasciculare* and related species.

In summary, the lichen family Pannariaceae includes genera with very different thalli, easily recognized by their morphology and anatomy and behavior to water availability, the collematoid and pannarioid thalli. We here wish :

1. to examine the phylogenetic relationships of the collematoid genera *Kroswia* and *Physma*, and to examine the phylogenetic relationships of the photobiont of these two taxa (both being lichenized with *Nostoc*);
2. to examine the phylogenetic relationships of the collematoid, pannarioid and tripartite thalli all across the family Pannariaceae, and to establish whether a photobiont switch can be associated with the transition towards from pannarioid thalli to collematoid thalli and vice versa;
3. to examine the phylogenetical position of an undescribed species with tripartite thallus, belonging to *Pannaria* s. l. (foliose species with a green algae in the thallus and developing squamulose cephalodia with *Nostoc* over its surface) and to assess the evolutionary significance of a thallus combining a green algae and a cyanobacteria.

## Materials and Methods

### Taxon Sampling

We assembled material belonging to the Pannariaceae from recent field trips in Madagascar (2008), Reunion Island (2008, 2009) and Thailand (2012). The 36 specimens used for molecular analysis are listed in Table 1. Identification of these collections is based on Jørgensen [27], [28], [38]–[44], Jørgensen & Schumm [45], Jørgensen & Sipman [46], Upreti et al. [47], Swinscow & Krog [48] and Verdon & Elix [49].

**Table 1:** Voucher table of the specimens used in the study, with the species names for the mycobiont, and the species names of the host for the photobiont, when available; the country of origin and the voucher information; GenBank accessions of the sequences.

Mycobiont species	Reference	Country of origin and voucher information	ITS	mtSSU	LSU	*RPB1*	cyanobacterial 16S
*Degelia durietzii* Arv. & D.J. Galloway	19	New Zealand		GQ259022	GQ258992	GQ259051	
*Degelia plumbea* (Lightf.) P.M. Jørg. & P. James	93 (ITS), 19	Norway (ITS), Portugal	AF429265	AY340491	AY340543	GQ259052	
*Erioderma verruculosum* Vain.	117	?		DQ972990	DQ973041	DQ973062	
*Fuscoderma applanatum* (D.J. Galloway & P. M. Jørg.) P.M. Jørg & D.J. Galloway	19	New Zealand		GQ259024	GQ258994	GQ259053	
*Fuscopannaria ahlneri* (P.M. Jørg.) P.M. Jørg.	118 (ITS), 19	Norway (ITS), South Korea	GU570097	GQ259025	GQ258995	GQ259054	
*Fuscopannaria confusa* (P.M. Jørg.) P.M. Jørg	118	Norway	GU570133	GU570043			
*Fuscopannaria ignobilis (*Anzi) P.M. Jørg.	119 (ITS), 117	?	HQ650673	DQ917416	DQ917417	DQ986839	
*Fuscopannaria leucosticta* (Tuck.) P.M. Jørg.	**NEW**	Reunion Island, R1123 (LG)	**KF704257**	**JX494238**	**JX494264**	**JX494284**	**KF704325**
*Fuscopannaria leucosticta* (Tuck.) P.M. Jørg.	93 (ITS), 19	USA	AF429277	DQ900630	DQ900640	GQ259055	
*Fuscopannaria mediterranea (*Tav.) P.M. Jørg.	118(ITS), 117	Norway (ITS)	GU570131	DQ917418	DQ917419		
*Fuscopannaria praetermissa* (Nyl.) P.M. Jørg.	118 (ITS), 19	Norway (ITS), Sweden	GU570108	GQ259026	GQ258996	GQ259056	
*Fuscopannaria praetermissa* (Nyl.) P.M. Jørg.	**NEW**	Reunion Island, R1060 (LG)	**KF704258**	**JX494239**		**JX494285**	**KF704346**
*Fuscopannaria sampaiana* (Tav.) P.M. Jørg.	118	Norway		GU570030			
*Joergensenia cephalodina* (Zahlbr.) Passo, S. Stenroos & Calvelo	96	Argentina	EU885308	EU885329			
*Kroswia crystallifera* P.M. Jørg.	**NEW**	Madagascar, M788 (LG)	**KF704254**	**JX494235**	**JX494261**	**JX494281**	**KF704343**
*Kroswia crystallifera* P.M. Jørg.	**NEW**	Reunion Island, R1055 (LG)	**KF704255**	**JX494236**	**JX494262**	**JX494282**	**KF704345**
*Kroswia crystallifera* P.M. Jørg.	**NEW**	Reunion Island, R1679 (LG)	**KF704256**	**JX494237**	**JX494263**	**JX494283**	**KF704344**
*Leciophysma furfurascens* (Nyl.) Gyeln.	19	Sweden		GQ259028	GQ258998	GQ259058	
*Leptogidium contortum* (Henssen) T. Sprib. & Muggia	34	Chile		JF938195			
*Leptogidium dendriscum* (Nyl.) Nyl.	34	USA, Alaska		JF938202	JF938143		
*Leptogium lichenoides* (L.) Zahlbr.	119 (ITS), 114	?	HQ650672	DQ923120	DQ917412	DQ917414	
*Pannaria athroophylla* (Stirt.) Elvebakk & Galloway	96	Argentina	EU885303	EU885325			
*Pannaria calophylla (*Müll. Arg.) Passo & Calvelo	96	Argentina	EU885296	EU885318			
*Pannaria conoplea* (Ach.) Bory	93 (ITS), 15	Norway (ITS)	AF429281		AY424209		
*Pannaria implexa* (Stirt.) Passo, Calvelo & Stenroos	95	Argentina	EU885311	EU885333			
*Pannaria lurida* (Mont.) Nyl.	**NEW**	Madagascar, M786 (LG)	**KF704248**	**JX494240**	**JX494265**	**KF704307**	
*Pannaria lurida* (Mont.) Nyl.	**NEW**	Reunion Island, R1033 (LG)	**KF704252**	**JX494247**	**JX494272**	**KF704311**	
*Pannaria lurida* (Mont.) Nyl.	**NEW**	Reunion Island, R1012 (LG)	**KF704253**	**JX494246**	**JX494271**	**KF704312**	
*Pannaria microphyllizans* (Nyl.) P.M. Jørg.	93 (ITS), 96	Australia (ITS), Argentina	AF429279	EU885322			
*Pannaria multifida* P.M. Jørg.	**NEW**	Reunion Island, R942 (LG)	**KF704249**	**JX494241**	**JX494266**	**KF704308**	
*Pannaria multifida* P.M. Jørg.	**NEW**	Reunion Island, R960 (LG)	**KF704251**	**JX494242**	**JX494267**	**KF704309**	
*Pannaria multifida* P.M. Jørg.	**NEW**	Reunion Island, R961 (LG)	**KF704250**	**JX494243**	**JX494268**	**KF704310**	
*Pannaria pallida* (Nyl.) Hue	96 (ITS, mtSSU), 87 (LSU)	Argentina	EU885301	EU885323	GQ927270		
*Pannaria rubiginella* P.M. Jørg.	19		GQ927269	GQ259037	GQ259007	GQ259074	
*Pannaria rubiginosa* (Thunb. ex. Ach.) Delise	19	Portugal	GQ927267	AY340513	AY340558	GQ259073	
*Pannaria rubiginosa* (Thunb. ex. Ach.) Delise	**NEW**	Reunion Island, R1008 (LG)	**KF704259**	**JX494244**	**JX494269**	**KF704313**	**KF704321**
*Pannaria rubiginosa* (Thunb. ex. Ach.) Delise	**NEW**	Reunion Island, R1126 (LG)	**KF704260**	**JX494249**	**JX494274**	**KF704315**	
*Pannaria rubiginosa* (Thunb. ex. Ach.) Delise	**NEW**	Reunion Island, R1011 (LG)	**KF704261**	**JX494245**	**JX494270**	**KF704314**	**KF704323**
*Pannaria* sp.	**NEW**	Thailand, T4 (LG)	**KF704247**	**KF704289**	**KF704290**	**KF704306**	**KF704333**
*Pannaria sphinctrina* (Mont.) Hue	96 (ITS, mtSSU), 87 (LSU)	Argentina	EU885302	EU885324	GQ927271		
*Pannaria tavaresii* P.M. Jørg.	96	Argentina	EU885294	EU885316			
*Pannaria* sp. (tripartite thallus)	**NEW**	Reunion Island, R969 (LG)	**KF704268**	**KF704286**		**KF704299**	**KF704341**
*Parmeliella appalachensis* P.M. Jørg.	117	?		DQ972992		DQ973064	
*Parmeliella borbonica* P.M. Jørg. & Schumm	**NEW**	Reunion Island, R1122 (LG)	**KF704271**	**JX494259**			**KF704320**
*Parmeliella brisbanensis* (C. Knight) P.M. Jørg. & D.J. Galloway	**NEW**	Thailand, T1 (LG)	**KF704246**	**KF704280**		**KF704292**	
*Parmeliella brisbanensis* (C. Knight) P.M. Jørg. & D.J. Galloway	**NEW**	Thailand, T3 (LG)	**KF704277**	**KF704281**		**KF704294**	**KF704351**
*Parmeliella brisbanensis* (C. Knight) P.M. Jørg. & D.J. Galloway	**NEW**	Thailand, T7 (LG)	**KF704276**	**KF704282**		**KF704295**	**KF704352**
*Parmeliella brisbanensis* (C. Knight) P.M. Jørg. & D.J. Galloway	**NEW**	Reunion Island, R1019 (LG)	**KF704278**	**JX494255**		**KF704296**	**KF704350**
*Parmeliella brisbanensis* (C. Knight) P.M. Jørg. & D.J. Galloway	**NEW**	Reunion Island, R1247 (LG)	**KF704262**	**JX494258**		**KF704297**	**KF704347**
*Parmeliella mariana* (Fr.) P.M. Jørg. & D.J. Galloway	**NEW**	Reunion Island, R974 (LG)	**KF704275**	**JX494256**		**KF704301**	**KF704330**
*Parmeliella miradorensis* Vain.	12	Spain, La Gomera		HQ268592		HQ268591	
*Parmeliella parvula* P.M. Jørg.	118	Norway	GU570099	GU570031			
*Parmeliella polyphyllina* P.M. Jørg.	**NEW**	Reunion Island, R1021 (LG)	**KF704265**	**JX494251**	**JX494276**	**KF704317**	**KF704327**
*Parmeliella polyphyllina* P.M. Jørg.	**NEW**	Reunion Island, R1058 (LG)	**KF704267**	**JX494252**	**JX494277**	**KF704319**	**KF704326**
*Parmeliella polyphyllina* P.M. Jørg.	**NEW**	Reunion Island, R1120 (LG)	**KF704266**	**JX494250**	**JX494275**	**KF704318**	
*Parmeliella* sp. (*mariana* gr.)	**NEW**	Thailand, T2 (LG)		**KF704283**		**KF704293**	**KF704348**
*Parmeliella* sp. (*mariana* gr.)	**NEW**	Thailand, T6 (LG)	**KF704279**	**KF704284**		**KF704304**	**KF704349**
*Parmeliella stylophora* (Vain.) P.M. Jørg.	**NEW**	Reunion Island, R979 (LG)	**KF704274**	**JX494257**		**KF704300**	**KF704331**
*Parmeliella triptophylla* (Ach.) Müll. Arg.	120(ITS), 19	Finland (ITS), Sweden	HM448807	AY652623	GQ259008	GQ259075	
*Parmeliella triptophylloides* P.M. Jørg.	**NEW**	Reunion Island, R965 (LG)	**KF704264**	**JX494253**	**JX494278**	**KF704316**	**KF704324**
*Peltigera aphthosa* (L.) Willd.	121, 122	Sweden (RPB1)	KC437624	AY340515	AF286759	DQ915598	
*Physma byrsaeum* (Ach.) Tuck.	19	Tahiti		GQ259039	GQ259010	GQ259077	
*Physma byrsaeum* (Ach.) Tuck.	**NEW**	Reunion Island, R2847 (LG)	**KF704272**	**JX494260**		**KF704303**	**KF704338**
*Physma byrsaeum* (Ach.) Tuck.	**NEW**	Reunion Island, R2 (LG)	**KF704273**	**KF704287**		**KF704302**	**KF704340**
*Physma byrsaeum* (Ach.) Tuck.	**NEW**	Reunion Island, R1121 (LG)	**KF704269**	**JX494254**		**KF704298**	**KF704337**
*Physma pseudoisidiatum* Aptroot & Sipman	19	USA		GQ259041	GQ259012		
*Physma radians* Vain.	19	Japan		GQ259040	GQ259011	GQ259078	
*Physma radians* Vain.	**NEW**	Thailand, T5 (LG)	**KF704270**	**KF704285**		**KF704305**	**KF704336**
*Placynthium nigrum* (Huds.) Gray	119 (ITS), 19	Sweden	HQ650699	AY340518	AF356674	GQ259079	
*Protopannaria pezizoides* (Weber ex. F.H. Wigg.) P.M. Jørg. & S. Ekman	93 (ITS), 19	Sweden	AF429271	AY340519	AY340561	GQ259081	
*Psoroma hypnorum* (Vahl.) Gray	93 (ITS), 19	Sweden	AF429272	AY340523	AY340565	GQ259085	
*Psoroma palaceum* (Fr.) Nyl.	96 (mtSSU), 87	Argentina	GQ927304	EU885327	GQ927305		
*Psorophorus pholidotus* Elvebakk & S.G. Hong	96 (mtSSU), 87	Argentina	EU885314	EU885336	GQ927289		
*Ramalodium succulentum* Nyl.	19	Australia		GQ259043	GQ259013	GQ259086	
*Staurolemma omphalarioides* (Anzi) P.M. Jørg. & Henssen	19	Norway		GQ259044	GQ259014		
*Staurolemma* sp.	**NEW**	Reunion Island, R982 (LG)	**KF704263**	**KF704288**	**KF704291**		**KF704329**
*Vahliella californica* (Tuck.) P.M. Jørg.	12	Canada, British Columbia		HQ268594		HQ268593	
*Vahliella leucophaea* (Vahl.) P.M. Jørg.	94 (ITS), 19	Sweden	AF429266	AY652621	DQ900642	GQ259090	
*Vahliella saubinetii* (Mont.) P.M. Jørg.	12	Croatia		HQ268602		HQ268601	
*Xanthopsoroma contextum* (Stirt.) Elvebakk	97	Argentina	EU885313	EU885335			
*Xanthopsoroma soccatum* (R. Br. ex Cromb.) Elvebakk	96, 87 (LSU)	Argentina	EU885315	EU885337	GQ927283		
							
**Cyanobacterial species**							
**(or host when applicable)**							
*Anabaena flos-aquae* Brébisson ex Bornet & Flauhault	Choi & Oh unpublished						DQ234825
*Anabaena oryzae* F.E. Fritsch	Mishra et al. unpublished	India					HM573456
*Anabaena vaginicola* F.E. Fristsch & Rich	Aghashariatmadari et al. unpublished	Iran					JN873351
*Blasia pusilla* 1 L.	Liaimer et al. unpublished	Norway					EU022724
*Blasia pusilla* 2 L.	Liaimer et al. unpublished	Norway					EU022708
*Blasia pusilla* 3 L.	Liaimer et al. unpublished	Norway					EU022728
*Blasia pusilla* 4 L.	Liaimer et al. unpublished	Norway					EU022717
*Chroococcus* sp.	123	Italy					FR798931
*Collema flaccidum* (Ach.) Ach.	124	Finland					DQ265959
*Collema nigrescens* (Huds.) DC.	125	USA California					JN847352
*Cycas revoluta* Thunb.	126	Italy					AM711533
*Fischerella muscicola* (Thuret) Gomont	127	strain PCC 7414					AF132788
*Fuscopannaria leucosticta* (Tuck.) P.M. Jørg.	**NEW**	Reunion Island, R1009 (LG)					**KF704322**
*Fuscopannaria leucosticta* (Tuck.) P.M. Jørg.	**NEW**	Reunion Island, R1124 (LG)					**KF704353**
*Gloeocapsa* sp.	128	strain PCC 73106					AB039000
*Gunnera prorepens* Hook. f.	126	New Zealand					AM711541
*Leptogium furfuraceum* 1 (Harm.) Sierk	125	USA California					JN847353
*Leptogium furfuraceum* 2 (Harm.) Sierk	129	USA, California					JQ007761
*Leptogium gelatinosum* (With.) J.R. Laundon	130	USA					DQ185232
*Leptogium lichenoides* 1 (L.) Zahlbr.	129	Scotland					JQ007765
*Leptogium lichenoides* 2 (L.) Zahlbr.	129	Scotland					JQ007766
*Leptogium palmatum* (Huds.) Mont.	125	USA Oregon					JN847344
*Leptogium pseudofurfuraceum* P.M. Jørg. & A.K. Wallace	125	USA California					JN847347
*Leptogium saturninum* (Dicks.) Nyl.	124	Finland					DQ265957
*Leptogium* sp.	**NEW**	Reunion Island, R2848 (LG)					**KF704328**
*Leptogium* sp.	**NEW**	Reunion Island, R2849 (LG)					**KF704335**
*Leptogium* sp.	**NEW**	Reunion Island, R2850 (LG)					**KF704334**
*Lobaria pulmonaria* 1 (L.) Hoffm.	125	USA Oregon					JN847345
*Lobaria pulmonaria* 2 (L.) Hoffm.	125	Norway					JN847357
*Lobaria scrobiculata* (Scop.) P. Gaertn.	129	Scotland					JQ007744
*Massalongia carnosa* (Dicks.) Körb.	130	USA					DQ185235
*Microcoleus chthonoplastes* Thur.	131						DQ460700
*Nephroma arcticum* (L.) Torss.	129	Finland					JQ007764
*Nephroma bellum* 1(Spreng.) Tuck.	120	Finland					HQ591510
*Nephroma bellum* 2 (Spreng.) Tuck.	120	Finland					HQ591518
*Nephroma laevigatum* Ach.	125	Norway					JN847359
*Nephroma parile* (Ach.) Ach.	120	Finland					HQ591521
*Nephroma resupinatum* (L.) Ach.	120	Finland					HQ591528
*Nephroma washingtoniense* Gyeln.	125	USA Oregon					JN847341
*Nodularia spumigena* Mertens	Beer et al. unpublished	USA Utah					FJ546713
*Nostoc commune* 1 Vaucher	132						AB088405
*Nostoc commune* 2 Vaucher	Gachon et al. unpublished	South Africa					HE974995
*Nostoc entophytum* Bornet & Flahault	Seo & Yokota unpublished						AB093490
*Nostoc linckia* (Roth) Bornet ex Bornet & Flahault	Seo & Yokota unpublished						AB074503
*Nostoc linckia* var. *arvense* C.B. Rhao	132						AB325907
*Nostoc muscorum* 1 C. Agardh ex Bornet & Flahault	133	Czech Republic					AJ630451
*Nostoc muscorum* 2 C. Agardh ex Bornet & Flahault	126	Czech Republic					AM711524
*Nostoc muscorum* 3 C. Agardh ex Bornet & Flahault	Mishra et al. unpublished	India					HM573462
*Nostoc muscorum* 4 C. Agardh ex Bornet & Flahault	126	Czech Republic					AM711523
*Nostoc muscorum* 5 C. Agardh ex Bornet & Flahault (soil)	130	France					DQ185254
*Nostoc punctiforme* (Kützing) Hariot Gunnera manicata	130	Germany					DQ185256
*Nostoc* sp. 1	Liaimer et al. unpublished	Norway					EU022737
*Nostoc* sp. 2	Suzuki et al. unpublished						GU062468
*Nostoc* sp. 3	Suzuki et al. unpublished						GU062469
*Nostoc* sp. 4(root of plant)	126	Italy					AM711532
*Nostoc* sp. 5	134	South Africa					AJ344563
*Nostoc* sp. 6	Liaimer et al. unpublished	Norway					EU022709
*Nostoc* sp. 7	Liaimer et al. unpublished	Norway					EU022729
*Nostoc* sp. 8	Mishra et al. unpublished	strain PCC 7120					HM573458
*Nostoc* sp. 9	132	strain PCC 7906					AB325908
*Nostoc* sp. 10	Liaimer et al. unpublished	Norway					EU022713
*Nostoc* sp. 11	126	Italy					AM711549
*Nostoc* sp. 12	135	Spain					HM623782
*Pannaria* aff. *leproloma* 1	17	Chile					EF174208
*Pannaria* aff. *leproloma* 2	17	Chile					EF174213
*Pannaria andina* 1 P.M. Jørg. & Sipman	17	Peru					EF174233
*Pannaria andina* 2 P.M. Jørg. & Sipman	17	Chile					EF536022
*Pannaria araneosa* (C. Bab.) Hue	17	New Zealand					EF174222
*Pannaria athroophylla* (Stirt.) Elvebakk & Galloway	17	Chile					EF174202
*Pannaria* cf. *allorhiza*	17	New Zealand					EF174206
*Pannaria conoplea* (Ach.) Bory	17	Norway					EF174221
*Pannaria durietzii* (P. James & Henssen) Elvebakk & D.J. Galloway	17	New Zealand					EF174227
*Pannaria elixii* P.M. Jørg. & D.J. Galloway	17	New Zealand					EF174230
*Pannaria fulvescens* (Mont.) Nyl.	17	New Zealand					EF174231
*Pannaria isabellina* 1 (Vain.) Elvebakk & Bjerke	17	Chile					EF174226
*Pannaria isabellina* 2 (Vain.) Elvebakk & Bjerke	17	Chile					EF174223
*Pannaria obscura* Müll. Arg.	17	Australia					EF174232
*Pannaria patagonica* (Malme) Elvebakk & D.J. Galloway	17	Chile					EF174204
*Pannaria rubiginella* P.M. Jørg.	17	Chile					EF536024
*Pannaria rubiginosa* (Thunb. ex. Ach.) Delise	17	Norway					EF174220
*Pannaria sphinctrina* Zahlbr.	17	Chile					EF174205
*Parmeliella triptophylla* (Ach.) Müll. Arg.	125	Norway					JN847361
*Peltigera aphthosa* (L.) Willd.	130	Switzerland					DQ185253
*Peltigera canina* 1 (L.) Willd.	130	USA					DQ185230
*Peltigera canina* 2 (L.) Willd.	Liaimer et al. unpublished	Norway					EU022726
*Peltigera didactyla* (With.) J.R. Laundon	130	Poland					DQ185245
*Peltigera evansiana* Gyeln.	129	USA, Oregon					JQ007784
*Peltigera leucophlebia* 1 (Nyl.) Gyeln.	136	Finland					FJ815321
*Peltigera leucophlebia* 2 (Nyl.) Gyeln.	129	Svalbard					JQ007783
*Peltigera malacea* (Ach.) Funch	137	Finland					EF102280
*Peltigera rufescens* 1(Weiss) Humb.	130	Germany					DQ185219
*Peltigera rufescens* 2 (Weiss) Humb.	130	Germany					DQ185215
*Peltigera scabrosa* Th. Fr.	Liaimer et al. unpublished	Norway					EU022727
*Peltigera* sp.	129	Argentina					JQ007785
*Physma byrsaeum* (Ach.) Tuck.	**NEW**	Reunion Island, R1 (LG)					**KF704342**
*Physma byrsaeum* (Ach.) Tuck.	**NEW**	Reunion Island, R2846 (LG)					**KF704339**
*Protopannaria pezizoides* (Weber ex. F.H. Wigg.) P.M. Jørg. & S. Ekman	124	Finland					DQ265953
*Pseudocyphellaria gilva* (Ach.) Malme	17	Chile					EF536023
*Pseudocyphellaria* sp.	125	USA California					JN847355
*Pseudocyphellaria* sp.	**NEW**	Reunion Island, R2332 (LG)					**KF704332**
*Scytonema* cf. *fritschii*	138	New Zealand					JN565281
*Scytonema hyalinum* 1 Gardner	139	USA					AF334698
*Scytonema hyalinum 2* Gardner	139	USA					AF334700
*Scytonema* sp. 1	140	Mexico					EU818953
*Scytonema* sp. 2	140	Costa Rica					EU818954
*Scytonema* sp.3	140	Costa Rica					EU818950
*Stereocaulon fronduliferum* I.M. Lamb.	124	New Zealand					DQ265951
*Stereocaulon ramulosum* Raeusch.	124	Hawaii					DQ265949
*Sticta limbata* (Sm.) Ach.	125	USA California					JN847351

Accessions in bold represent newly sequenced specimens.

### Molecular Data

Well-preserved lichen specimens lacking any visible symptoms of fungal infection were selected for DNA isolation. Extraction of DNA followed the protocol of Cubero et al. [50]. We sequenced the ribosomal nuclear loci ITS, using primers ITS1F [51] and ITS4 [52], and LSU with primers LR0R [53] and either LR7 [53] or LIC2044 [54], the mitochondrial ribosomal locus mtSSU, using primers SSU1 and SSU3R [55], and part of the protein-coding gene *RPB1* with RPB1AF [56] and RPB1CR [57]. We sequenced the 16S ribosomal region of the *Nostoc* symbiont of 25 of this set of Pannariaceae as well as 2 additional *Fuscopannaria leucosticta*, 2 additional *Physma* and 4 from two other genera (*Leptogium* and *Pseudocyphellaria*) belonging to the Peltigerales, using the two primer pairs fD1 [58]–revAL [17] and f712 [59]–rD1 [58]. Amplicons were sequenced by Macrogen**©** or by the GIGA technology platform of the University of Liège.

### Sequences Editing and Alignment

Sequence fragments were assembled with Sequencher version 4.9 (Gene Codes Corporation, Ann Arbor, Michigan). Sequences were subjected to megaBLAST searches [60] to detect potential contaminations. Sequences were aligned manually using MacClade version 4.08 [61]. Ambiguous regions were delimited manually and excluded from the analyses. Substitutions and indels in ITS1 and ITS2 were so numerous that no unambiguous alignment could be realized; therefore ITS sequences were reduced to the less variable 5.8S portion.

### Concatenation and Partitioning

Congruence of the four fungal loci was assessed by the comparison of single-locus phylogenetic trees produced with RAxML HPC2 version 7.2.8 [62]–[63] as implemented on the CIPRES portal [64], looking for the best ML tree and bootstrapping with 1000 pseudoreplicates in the same run, using GTRCAT model and the default settings. No significant conflict with bootstrap values (BS) >70 was detected and we therefore concatenated the different loci. As several species are represented by sequences obtained from specimens collected in the different parts of the world, mostly with ITS, we further assembled a 3 loci dataset excluding ITS. We thus produced three matrices, two for a large sampling of the Pannariaceae including our target taxa (*Kroswia, Physma* and the undescribed species with a tripartite thallus), including the four loci 5.8S, mtSSU, LSU and RPB1 or including only the latter three, and one with the *Nostoc* 16S data.

For the concatenated analysis of the four loci, we partitioned the data in different subsets to optimize likelikood. We used PartitionFinder [65] to choose the best partition and determine the best models for the different subsets. We used BIC as the criterion to define the best partition, and compared all models implementable in MrBayes [66]. The partition tested for the analysis on the four loci was composed of 6 subsets: *RPB1*, 1^st^ codon position, *RPB1*, 2^nd^ codon position, *RPB1* 3^rd^ codon position, mtSSU, LSU, 5.8S. For the 16S analysis on *Nostoc*, we used MrModelTest version 2.3 [67] to determine the best model.

### Maximum Likelihood and Bayesian Phylogenetical Analyses

For each matrix, we produced the best likelihood tree and bootstrapped for 1000 pseudoreplicates in the same run using RAxML version 7.4.2 [62]–[63] with the default settings and the GTRCAT model. We further ran a Bayesian analysis using MrBayes version 3.1.2 [66]. Each analysis consisted of 2 runs of 3 heated chains and 1 cold one. We assessed the convergence using Tracer version 1.5 [68] and stopped the runs after checking with AWYT [69] that convergence was reached for each run and that tree topologies have been sampled in proportion of their true posterior probability distribution. The analysis for the family Pannariaceae was stopped after 15×10^6^ generations, the analysis on *Nostoc* 16S after 37×10^6^ generations.

### Ancestral State Reconstruction

We reconstructed ancestral character states using SIMMAP version 1.5.2 [70], with default settings, on the consensus Bayesian tree produced by the MrBayes analysis on the Pannariaceae 4 loci concatenated dataset, as well as on a subset of 20 trees (10 from each run of the Bayesian analysis) and with Mesquite version 2.75 [71]–[72] using the likelihood parameters and the default settings, calculating the average probabilities of the ancestral states based on the same subset of 20 trees.

We also used BayesTraits version 1.0 [73] on a set of 2 trees: the best tree produced by the ML analysis on the Pannariaceae 4 loci concatenated dataset and on the best tree of the concatenated analysis without 5.8S, as they were slightly different, to constrain some branches (ancestors) to be to a certain state. We compared the harmonic mean of the iterations, which is an approximation of the marginal likelihood of the model, calculating the Bayes Factor, which is twice the difference of likelihood between the models, with each state of ancestor, to see which state of the ancestor leads to the best likelihood of the model. A positive Bayes Factor suggests that the first character state tested has a better likelihood than the second one, and a Bayes Factor above 2 is considered significant (Bayestraits Manual, available at http://www.evolution.rdg.ac.uk/BayesTraits.html). We used reversible jump and a gamma hyperprior whose mean and variance vary between 0 and 10. We ran the program for 50×10^6^ iterations for each constrained state. The character reconstructed was the type of thallus, and the character states considered were tripartite, pannarioid bipartite and collematoid bipartite.

### Topological Tests

We tested different tree topologies on the concatenated dataset of 4 loci for the Pannariaceae. We generated 8 constrained best trees with RAxML, with the same settings as above, and using the following constraints: (1) the 3 accessions of *Kroswia* forming a monophyletic group; (2) *Kroswia* as a monophyletic group basal to a group formed by *Fuscopannaria ahlneri, F. confusa, F. leucosticta* and *F. praetermissa*; (3) *Kroswia* as a monophyletic group basal to all accessions of *Fuscopannaria* except *F. sampaiana*; (4) all accessions of *Fuscopannaria* except *F. sampaiana* as basal to the *Physma* clade (which includes *Parmeliella borbonica,* the *Parmeliella mariana* group and the tripartite R969 in addition to all accessions of *Physma*) and the *Pannaria* clade (all *Pannaria* except the tripartite R969), to compare our results with the topology retrieved in Wedin et al. [19] and Spribille & Muggia [10]; (5) the tripartite species annotated as the tripartite R969 as basal to a group formed by all accessions of *Parmeliella mariana* group and *Physma* resolved in the same clade; (6) all accessions of *Physma* as basal to all accessions of *Parmeliella mariana* group and the tripartite R969 in the same clade; (7) *Parmeliella borbonica* basal to all accessions of *Physma*; (8) all accessions of *Physma* basal to all accessions of *Parmeliella mariana* group including *Parmeliella borbonica* in the same clade.

We computed the likelihood of 100 trees (the best constrained tree, the best unconstrained tree and a random sample of 98 bootstrap replicate trees from the unconstrained analysis), estimating parameters on a NJ tree, using an HKY model with a gamma rate of heterogeneity and 4 gamma categories (parameters choice and methodology suggested by [74]). We performed the 1sKH test [75]–[77], the SH test [75] and the ELW test [78] on the constrained tree using TreePuzzle v. 5.2. [79]. Due to its very low power (see for instance [74]), we did not consider the results of the SH test.

## Results

### Molecular Data

We amplified ITS, mtSSU and *RPB1* for all 36 selected specimens, except one for *RPB1*. We amplified LSU for 21 specimens, all 15 negative results being resolved in a single clade comprising all accessions of *Physma*, the *Parmeliella mariana* gr. (*P. brisbanensis, P. mariana* and *P. stylophora*), *Parmeliella borbonica* and the undescribed tripartite ‘*Pannaria’* R969 (here annotated the tripartite R969). Wedin et al. [19] could amplify the LSU loci for three species of *Physma*, but, for unknown reasons, all our attempts to amplify LSU for this clade failed.

### Matrix Assemblage and Concatenation

For the analysis on the Pannariaceae mycobiont, we could include the following newly sequenced specimens: 21 specimens with all 4 loci, 14 with 3 loci (lacking LSU) and 1 specimen with 2 loci (lacking LSU and *RPB1*). We added 46 taxa retrieved from GenBank to complete our sampling, 39 members of the Pannariaceae, and 7 outgroup taxa all belonging to the Peltigerales (3 Vahliellaceae, 1 Collemataceae, 1 Placynthiaceae, 1 Peltigeraceae). Those included either the 4 loci or a subset of them. Detailed information can be found in table 1. For the 16S dataset on *Nostoc*, we produced 36 new sequences; we added 93 *Nostoc* sequences retrieved from GenBank, chosen either on the phylogenetic position of their fungal partner or their nucleotide similarity to our sequences, based on megaBLAST searches [60], and 14 outgroup sequences, belonging to other genera, to complete our sampling.

### Partitioning and Model Selection

For the analysis on the Pannariaceae mycobiont, PartitionFinder divided the partition in 4 subsets: one composed of *RPB1* 1^st^ and 2^nd^ codon positions with LSU, one with mtSSU only, one with 5.8S only and one with *RPB1* 3^rd^ codon position only. For the first subset, the model selected was GTR+I+G, as well as for mtSSU and *RPB1* 3^rd^ codon position; for 5.8S, the model selected was K80+I+G. For the analysis on the *Nostoc* 16S dataset, the model selected was GTR+I+G.

### Phylogenetic Analyses

**Figure 1:**
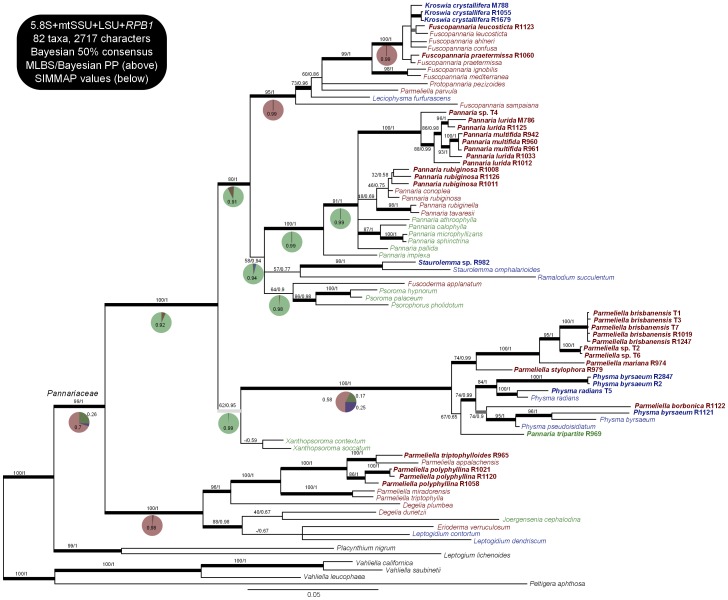
Phylogenetic relationships in the family Pannariaceae, based on the 50% Bayesian consensus tree of the analysis on 4 loci (5.8S, LSU, mtSSU, *RPB1*). Values above branches represent ML bootstrap and Bayesian PP values, respectively. Colors in the taxa names and pie charts represent the type of the thallus: in green tripartite thalli, in red pannarioid thalli and in blue collematoid thalli. Pie charts refer to the SIMMAP analysis on this tree. Names in bold are those for which DNA sequences were produced for this study. Thick black branches have MLBS >70 and Bayesian pp>0.95, dark grey branches have MLBS >70 but pp<0.95 and light grey branches have pp>0.95 but MLBS<70.

**Figure 2:**
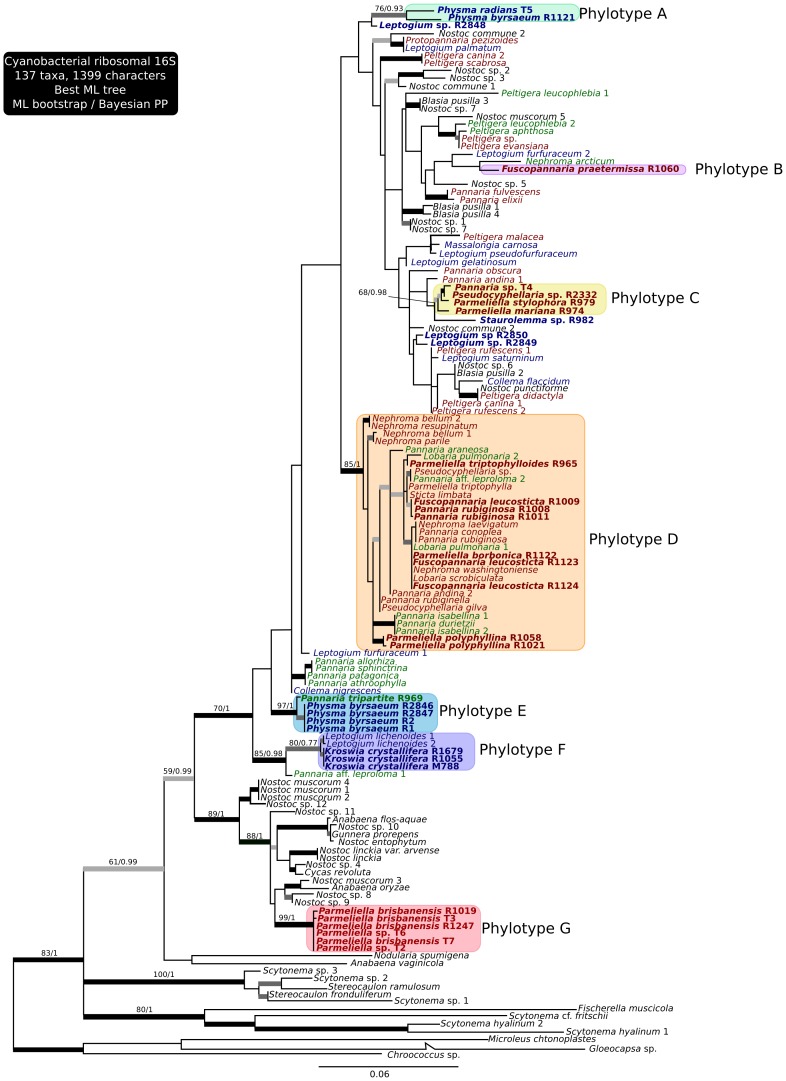
Phylogenetic relationships in the genus *Nostoc*, based on the best ML tree of the analysis on the 16S dataset. Values above branches represent ML bootstrap and Bayesian PP values, respectively. Names in bold are those for which DNA sequences were produced for this study. Color boxes represent phylotypes containing our sequences and defined by well-supported monophyletic groups. Colors in the taxa names represent the type of the thallus containing the *Nostoc*: in green tripartite thalli, in red pannarioid thalli and in blue collematoid thalli. Taxa names refer to the host of the *Nostoc* symbionts, when available. Thick black branches have MLBS >70 and Bayesian pp>0.95, dark grey branches have MLBS >70 but pp<0.95 and light grey branches have pp>0.95 but MLBS<70.

The 50% Bayesian consensus tree of the analysis of the Pannariaceae mycobiont dataset comprizing 4 loci is presented in Figure 1, with the bootstrap values of the ML analysis and the Bayesian PP values written above the branches. The same consensus tree obtained with the 3 loci dataset is available in the Supplementary Material (Figure S1). The 50% Bayesian consensus tree of the analysis of the *Nostoc* 16S dataset is presented in Figure 2, with the bootstrap values of the ML analysis and the Bayesian PP values written above the branches.

### Phylogeny of the Family Pannariaceae (Fig. 1)

Topology of the family.

The analysis of the 3 and 4 loci datasets yielded the same topology, albeit with less support for some branches for the former; as expected the 5.8S loci provides an interesting resolution power to discriminate branches at the generic and infrageneric level. We retrieved the Pannariaceae as a monophyletic group, divided into two strongly supported clades: the first one includes all *Parmeliella* accessions, incl. the genus type *P. triptophylla*, except for the *P. mariana* group and *P. borbonica* which are resolved with strong support in the other clade. The so-called *Parmeliella* s. str. clade further includes *Degelia* (here resolved as polyphyletic, as already detected by Wedin et al. [19]), *Erioderma*, *Leptogidium* and the monotypic *Joergensenia* which represents the only tripartite species in this clade. The second clade can be divided into three groups: (1) the first one is not supported in ML optimization but gets a PP = 0.95 in the Bayesian analysis; it is composed of *Xanthopsoroma*, *Physma*, the *Parmeliella mariana* group, *Parmeliella borbonica* and the tripartite species R969, and will be referred to as the *Physma* group; (2) a group not supported in ML optimization but getting a PP = 0.94 in Bayesian analysis, composed of *Pannaria*, *Staurolemma*, *Ramalodium*, *Fuscoderma*, *Psoroma* and *Psorophorus*, that will be referred to as the *Pannaria* group; and finally (3) a group composed of *Fuscopannaria*, *Kroswia*, *Protopannaria*, *Leciophysma* and *Parmeliella parvula*, that will be referred to as the *Fuscopannaria* group.

Wedin et al. [19] and Spribille and Muggia [10] retrieved the *Parmeliella* s. str. group, the *Pannaria* group and the *Fuscopannaria* group with similar topology as ours. However, in their studies, their single or multiple accessions of *Physma* is or are nested within the *Pannaria* group. With our dataset, which includes a larger sampling of *Physma* and representatives of the closely related *Parmeliella mariana* gr., *P. borbonica* and the tripartite R969, the hypothesis of the whole *Physma* group nested in the *Pannaria* group and the *Fuscopannaria* group as basal is strongly rejected by two topological tests (ELW and 1sKH tests; see table 2).

**Table 2:** Topology tests.

Constraint	logL best tree	diff. with unconstrained	1sKH test	ELW test
*Kroswia* monophyletic	−19700.43	2.77	0.312	0.0816
*Kroswia* out of *F. leucosticta* group	−19711.34	13.68	0.145	0.0239
*Kroswia* out of *Fuscopannaria* s. str.	−19741.75	44.09	**0.002**	**0**
*Physma* group in *Pannaria* group, *Fuscopannaria* group basal	−19730.55	32.89	**0.019**	**0.011**
R969 basal out of *Physma/Parmeliella mariana* group	−19701	3.34	0.299	0.0816
*Physma* basal to R969/*Parmeliella mariana* group	−19731.4	33.75	**0.007**	**0**
R1122 basal to *Physma*	−19703.25	5.59	0.165	0.041
R1122 basal to *P. mariana* group; *Physma* outside	−19707.95	10.29	0.094	0.018

Likelihood values of the best trees and results of the 1sKH test and ELW test on the different constraints on the topology of the tree. Results in bold significantly reject the concerned topologies.

### Monophyly of Several Genera

Our accessions of *Kroswia crystallifera* (the type species of the genus; [27]) gathered in Madagascar and Reunion are not resolved as a monophyletic group: they are nested within *Fuscopannaria*, and closely related to its type species *F. leucosticta* [38]. Even with the exclusion of species now referred to *Vahliella* [12], [80], the genus *Fuscopannaria* is not resolved as monophyletic, unless *F. sampaiana* is excluded and *Kroswia crystallifera* included. Two strongly supported clades can be distinguished if the genus is so recircumscribed: one with *F. ignobilis* and *F. mediterranea* and the other with the type species and *Kroswia crystallifera*.

*Pannaria* is resolved as a diverse but nevertheless well-supported genus, including several tripartite species formally placed in the genus *Psoroma* and which were transferred to *Pannaria* following the detailed studies by Elvebakk [81]–[84], Elvebakk & Bjerke [85], Elvebakk & Galloway [86] and Elvebakk et al. [17]. Interestingly, our single accession of the tripartite *Pannaria*-like R969 is not resolved amongst other tripartite *Pannaria* but within the *Physma* clade with strong support. It therefore appears that the tripartite *Pannaria*-like species are more diverse than expected and that the tripartite habit is widespread amongst the Pannariaceae, being absent only in the *Fuscopannaria* group. Two recently described and tripartite genera *Xanthopsoroma* and *Psorophorus*, segregated from *Psoroma* [87], are retrieved as a part of the *Physma* gr. with support only in the Bayesian analysis for the former, and as sister to *Psoroma* s. str. in the *Pannaria* group for the latter.

*Parmeliella* (type species: *P. triptophylla*) is a well-supported monophyletic group if the *Parmeliella mariana* gr., *Parmeliella borbonica* and *P. parvula* are excluded. The latter is resolved with strong support within the *Fuscopannaria* gr. whilst the others are resolved within the *Physma* group, on a long and strongly supported branch. Further, *P. borbonica* appears nested inside *Physma*, which is therefore paraphyletic.

### *Nostoc* Phylogeny (Fig. 2)

We defined phylotypes (A to G) on the *Nostoc* tree based on well-supported monophyletic groups containing sequences from our representatives of the Pannariaceae family. All our sequences are part of *Nostoc* clade 2 (*sensu* [59], [88]) except phylotype G, which seems related to *Nostoc* clade 3 *sensu* Svenning et al. [59].

There is no evidence suggesting coevolution or cospeciation events between the mycobiont and the photobiont. The phylogeny of *Nostoc* involved in the lichen symbiosis does not match the phylogeny of the Pannariaceae.

### Topological Uncertainties (Table 2)

he tests do not reject the monophyly of *Kroswia*, either its position outside of the polytomy including *i.a. Fuscopannaria leucosticta* and *F. praetermissa*, although the difference of likelihood with the best unconstrained tree is relatively high (13.68). However, the position of *Kroswia* outside of *Fuscopannaria* s. str. (including *F. mediterranea* and *F. ignobilis*) is significantly rejected by the ELW and 1sKH tests. Therefore *Kroswia crystallifera* should be considered as part of *Fuscopannaria*.

Concerning the position of the tripartite R969, the topological tests do not reject its position at the base of the *Physma* group as a whole. However, its position at the base of the *Parmeliella mariana* gr., with *Physma* basal to both of them, is significantly rejected by the ELW and 1sKH tests.

Concerning the position of *Parmeliella borbonica*, the topological tests do not reject its position neither as basal to *Physma*, nor as basal to the *Parmeliella mariana* gr., with *Physma* basal to both of them, although the difference of likelihood for the latter case is relatively high (10.29). We consider that the weak resolution of the test regarding the position of *Parmeliella borbonica* might be due to a large amount of missing data as only 2 loci are available for this accession, reducing its impact on the likelihood of the trees. More material should therefore be studied before the taxonomic status of *P. borbonica* can be revised.

As commented above, we also tested the topology proposed by Wedin & al. [19] and Spribille & Muggia [10] where their accessions of *Physma* are resolved within the *Pannaria* gr. Such a topology is rejected on our dataset by the ELW and 1sKH tests.

### Reconstruction of Ancestral States (Fig. 1, Table 3)

Results of the SIMMAP reconstructions on the Bayesian consensus tree are shown in pie charts on Figure 1. Results of the BayesTraits and Mesquite reconstructions, as well as the SIMMAP reconstruction on 20 trees are shown in table 3.

**Table 3:** Reconstruction of ancestral states.

Node	SB	S20	M	BF[T>P]	BF[T>C]
*F. leucosticta*+*F. praetermissa*	P = 0.99	P = 0.99	P = 0.99		
*Fuscopannaria* s. str. (incl. *F. ignobilis*, w/o *F. sampaiana*)	P = 0.99	P = 0.99	P = 0.99		
*Fuscopannaria* gr. (incl. *F. sampaiana*)	P = 0.99	P = 0.97	P = 0.73		
genus *Pannaria*	T = 0.99	T = 0.98	T = 0.91	9.66	
genus *Pannaria* w/o *P. implexum*	T = 0.99	T = 0.8	T = 0.84		
*Psoroma*+*Psorophorus*+*Fuscoderma*	T = 0.98	T = 0.93	T = 0.83		
*Pannaria* group (incl. *Psoroma*, *Staurolemma* etc.)	T = 0.94	T = 0.86	T = 0.81		
*Fuscopannaria*+*Pannaria*	T = 0.91	T = 0.84	T = 0.77	1.4	
*Physma*+*Parmeliella mariana* gr.	P = 0.58	P = 0.5	P = 0.39	0.32	3.94
*Physma*+*Parmeliella mariana* gr.+*Xanthopsoroma*	T = 0.99	T = 0.99	T = 0.91	11.7	8.7
*Fuscopannaria*+*Pannaria*+*Physma*	T = 0.92	T = 0.89	T = 0.815	1.06	
*Parmeliella* s. str. gr. (incl. *Erioderma* etc.)	P = 0.98	P = 0.99	P = 0.87		
family Pannariaceae	P = 0.7	P = 0.71	P = 0.46		

T = tripartite, P = pannarioid, C = collematoid. SB = SIMMAP results on the 50% consensus Bayesian tree, S20 = SIMMAP results on the subset of 20 trees, M = Mesquite results, BF = Bayes Factor of the BayesTraits analysis, T>P = Tripartite rather than pannarioid ancestor, T>C = Tripartite rather than collematoid ancestor.

Even though the probability values can vary quite widely from a reconstruction method to the other, the same ancestral character state is recovered for most branches.

For the *Fuscopannaria* group, a pannarioid ancestor is strongly supported, incl. for the *Fuscopannaria* s. str. clade (all *Fuscopannaria* except for *F. sampaiana*). Within the *Pannaria* group, two deep nodes are recovered with a tripartite ancestor (the unresolved clade with all accessions of *Pannaria*, and the clade including *Fuscoderma, Psoroma* and *Psorophorus*) as well as the node supporting the whole group. The node supporting both groups (the *Fuscopannaria* and the *Pannaria* gr.) also has tripartite thallus as the most likely ancestral type. For the clade comprizing *Physma,* the *Parmeliella mariana* gr., *P. borbonica* and the tripartite R969, reconstructions favor a pannarioid ancestor without much support, except the Bayes Factor that slightly favors a tripartite ancestor. However, for the whole group and thus including both accessions of *Xanthopsoroma*, reconstructions recover a tripartite ancestor with strong support. The node supporting the three groups (*Fuscopannaria*-, *Pannaria*-, and *Physma*-group) has most likely a tripartite thallus, as recovered by all four methods. The *Parmeliella* s. str. group most probably had a pannarioid ancestor, as well as the family Pannariaceae.

## Discussion

### *Nostoc* from Collematoid and Pannarioid Thalli (Fig. 2)

**Figure 3:**
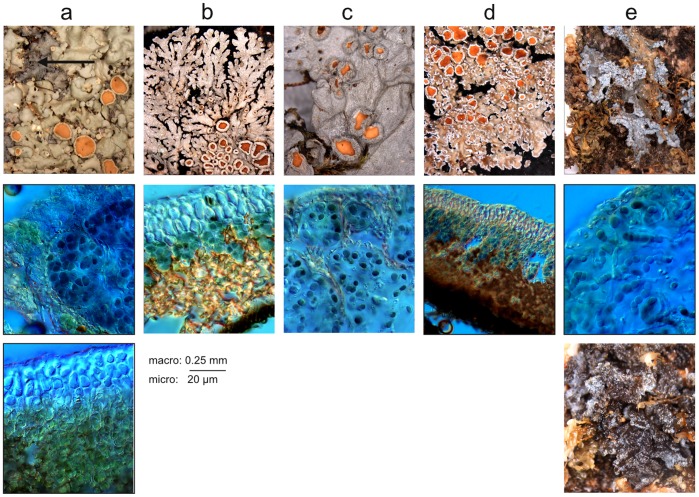
Selected pictures of studied Pannariaceae. Column, from left to right: a: tripartite R969, b: pannarioid *Parmeliella mariana*, c: collematoid *Physma byrsaeum*, d: pannarioid *Fuscopannaria leucosticta*, e: collematoid *Kroswia crystallifera*. Top row: macroscopic pictures showing the general aspect of the thallus; arrow point to cephalodia. Middle row: microscopic pictures showing the position of the *Nostoc* cells inside the thallus. Bottom row, left: Microscopic picture showing the position of the green algal cells in the thallus; right: macroscopic picture showing the aspect of *Kroswia* when wet.

Thalli belonging to the collematoid or pannarioid types never share the same *Nostoc* phylotype. Phylotypes A, E and F only contain symbionts from collematoid thalli. Moreover phylotype F also contains symbionts associated with the lichen genus *Leptogium*, a typical representative of the collematoid type, these accessions being resolved in a strongly supported clade together with the *Kroswia* symbionts. Phylotype E includes the photobiont of several *Physma* accessions together with that of the cephalodia of the tripartite R969, and these cephalodia have the same homoiomerous structure as the thallus of *Physma byrsaeum* (Fig. 3a, c).

Phylotypes B, C, D and G only contain symbionts from pannarioid thalli. Phylotype B which contains the photobiont of our accession of the terricolous *Fuscopannaria praetermissa* is closely related to sequences from terricolous-muscicolous *Nephroma arcticum* photobionts whereas phylotypes C and D contain *Nostoc* sequences from epiphytic *Lobaria, Nephroma* and *Pseudocyphellaria*, along with our accessions of epiphytic Pannariaceae with pannarioid thalli. This confirms that *Nostoc* from epiphytic heteroimerous thalli cluster together, although they group in a polyphyletic assemblage of different phylotypes [17], [89], [90]. These data strongly suggest that many pannarioid thalli share *Nostoc* strains between them and with other representatives of the Peltigerales that also have *Nostoc* in a well-defined thin layer. Furthermore collematoid thalli can share *Nostoc* with representatives of the Collemataceae that also have *Nostoc* chains throughout their thallus.

These results strongly suggest that the thallus type (collematoid versus pannarioid), and the organization of the *Nostoc* cells inside it, depend on the phylotype of the *Nostoc* with which the mycobiont associates. Therefore, it seems that in the family Pannariaceae, the *Nostoc* associated with the mycobiont would have more impact on the morphology of the thallus formed than the phylogenetic origin of the mycobiont. The corollary might be true as well, the *Nostoc* selection by the mycobiont is more affected by the morphological and ecophysiological characteristics of the association than by the phylogenetic position of the mycobiont. Extracellular polysaccharides substances (EPS) produced by many bacterial lineages, incl. cyanobacteria, are involved in the physiological and ecological characteristics of those organisms [91]; in *Nostoc*, the biochemistry and structure of the dense sheath of glycan strongly participate in the dessication tolerance of *Nostoc commune* [92]. Although no clear evidence is available, we suspect that variations in the glycan sheath characteristics amongst the various strains of *Nostoc* involved in the lichenization events within the Pannariaceae drive the differences between the collematoid and the pannarioid thallus types.

### Occurrence of Collematoid Thalli All across the Pannariaceae (Fig. 1)

We found collematoid thalli in the four main groups of the family. *Kroswia* and *Leciophysma* appear as part of the *Fuscopannaria* group, *Kroswia* being nested within *Fuscopannaria* s. str., excluding *F. sampaiana*; *Staurolemma* and *Ramalodium* are part of the *Pannaria* group and *Pannaria santessonii* was described as a collematoid thallus species; *Physma* is in the *Physma* group, along several taxa with pannarioid thalli; and finally *Leptogidium* is part of the *Parmeliella* s. str. group. These results suggest that thalli switched from pannarioid type to collematoid and possibly vice versa several times along the evolutionary history of the family.

These results also suggest that the thallus type organized by the association between a mycobiont and a photobiont is primarly driven by the identity of the latter, the *Nostoc* phylotype with which it associates rather than by the phylogenetic identity of the mycobiont. Indeed, unlike the original assumption that all collematoid thalli were part of the Collemataceae and all pannarioid thalli were part of the Pannariaceae, many collematoid thalli are actually members of the Pannariaceae, as already detected by Wedin et al. [19] and Otálora et al. [35]. Moreover, they do not form a monophyletic group inside the Pannariaceae, but are present all across the family, suggesting the absence of phylogenetic pattern of the mycobiont related to the collematoid morphological and anatomical thallus type.

### Evidence for Coincidence between Photobiont Switch and Change of Thallus Type

The most spectacular and straightforward example lies with the type species of *Kroswia* which is nested inside *Fuscopannaria* s. str.: it exhibits a drastic change of morphology (see figure 3d–e) of the thallus (all representatives of this genus so far have typical pannarioid thalli), and it associates with a *Nostoc* phylotype (phylotype F) that is totally different from the one associating with the closely related *Fuscopannaria leucosticta* (phylotype D). Moreover, phylotype F has also been found associated with the typically collematoid *Leptogium lichenoides*. The duo *Kroswia/Fuscopannaria* thus provides the best example of the influence of the *Nostoc* on the shape of the thallus. Actually, *K. crystallifera* is a species of *Fuscopannaria* with little genetic divergence with its related species such as *F. leucosticta* and *F. praetermissa*; this divergence however precludes any assumption that it could be considered as a photomorph of one of them. Its thallus is dramatically different because it switched to a different *Nostoc*, one that triggers the collematoid format for the thallus. Jørgensen [24], when studying the apothecia characters of the other species assigned to that genus (*K. gemmascens*), concluded that “the characters of the hymenium and the chemistry of the thallus certainly place it close to *Fuscopannaria* (…)”. Quite interestingly another photobiont switch can be postulated in that group as the phylogenetic position of *Moelleropsis nebulosa* as sister to *F. leucosticta* has been retrieved by Ekman & Jørgensen [93] and more recently announced as confirmed [94]. This species exhibits granulose thalli with clusters of *Nostoc* interwoven and covered by short-celled hyphae and very much different from the pannarioid thallus type, and thus most probably associated with a different *Nostoc* phylotype.

### Occurrence of Tripartite Thalli All across the Pannariaceae (Fig. 1)

We could detect tripartite thalli in all main groups within the family, except in the *Fuscopannaria* group. This absence might be caused by incomplete sampling as the only tripartite species known in *Fuscopannaria* (*F. viridescens*, associated with a green algae and producing cephalodia; [95]) as well as both species of *Degeliella* (forming tripartite thalli; [42]) could not be included in our dataset. *Psoroma*, *Psorophorus* and the tripartite representatives of *Pannaria* are resolved in the *Pannaria* group, *Xanthopsoroma* and the tripartite R969 belong to the *Physma* group, and the characteristic *Joergensenia* is included in the *Parmeliella* group. Until the seminal papers by Elvebakk & Galloway [86] and Passo et al. [96], all tripartite Pannariaceae were assigned to a single genus (*Psoroma*) assumed to form a monophyletic group. Within the three main groups of the Pannariaceae where they are resolved, the species with tripartite thalli are mixed up with species with bipartite thalli, mainly of pannarioid type but also with collematoid type. These results suggest that several times through the history of the family, mycobionts switched from a tripartite to a bipartite thallus or vice versa.

### Evidence for Cephalodia Emancipation

Switches from a tripartite to a bipartite thallus may involve the cephalodia and their emancipation from their green algae-containing thalli. Although cephalodia are usually associated with rather small, firmly attached, or even included, structures, there are many examples of tripartite *Pannaria* and *Psoroma* in which cephalodia are large and easily detached, or proliferating and developing large squamules that can be easily detached from their “host” thalli (examples in [17], [81], [97], [98]). The cephalodia of the tripartite R969 start their development as modest blue gray squamules over the thallus, but eventually grow up to 0.7 cm across and develop a foliose habit with denticulate to deeply lobulate margin (see figure 3a).

More interestingly, the *Nostoc* photobiont in several accessions of *Physma byrsaeum* (annotated R1, R2, R2846 and R2847; phylotype E) is very closely related to the one found in the cephalodia of the tripartite R969. As the latter is basal to the clade containing all accessions of *Physma*, it can be postulated that several species belonging to this genus arose from cephalodia emancipation from their common ancestor. Indeed, the common ancestor of the whole *Physma* clade is recovered as producing tripartite thallus. Furthermore, the disposition of the *Nostoc* cells inside the cephalodia of R969 is similar to the one inside *Physma* thalli (see figure 3a–c): they are enclosed in ellipsoid chambers delimited by medulla hyphae, these structures being responsible for the maculate upper surface of thalli (*Physma*) or cepahodia (R969).

Besides the tripartite R969, the clade included both accessions of the recently described genus *Xanthopsoroma* [87], which also develops tripartite thalli, with a green algae as the main photobiont and *Nostoc* included in cephalodia. The three species recognized within the *Parmeliella mariana* gr. may have arisen from cephalodia emancipation of their common tripartite ancestor or from a photobiont switch from a *Physma* ancestor. Quite interestingly, the pannarioid *Parmeliella borbonica*, nested within *Physma*, is associated with phylotype D of *Nostoc*, shared by most accessions of the *Pannaria* and *Parmeliella* s. str. groups (as well as other distantly related species of the Peltigerales), and not phylotypes C or G, chosen by all our accessions of its closely related species of the *Parmeliella mariana* gr. When excluding both accessions of *Xanthopsoroma*, the *Physma* gr. is a well-supported clade on a long branch and includes a tripartite species, species with pannarioid as well as collematoid thalli. The long branch may indicate that our sampling is too scarce and geographically too restricted. However, as both *Physma* and the *Parmeliella mariana* gr. have a pantropical distribution, we can confidently assume it would not collapse in future studies.

**Figure 4.Scheme:**
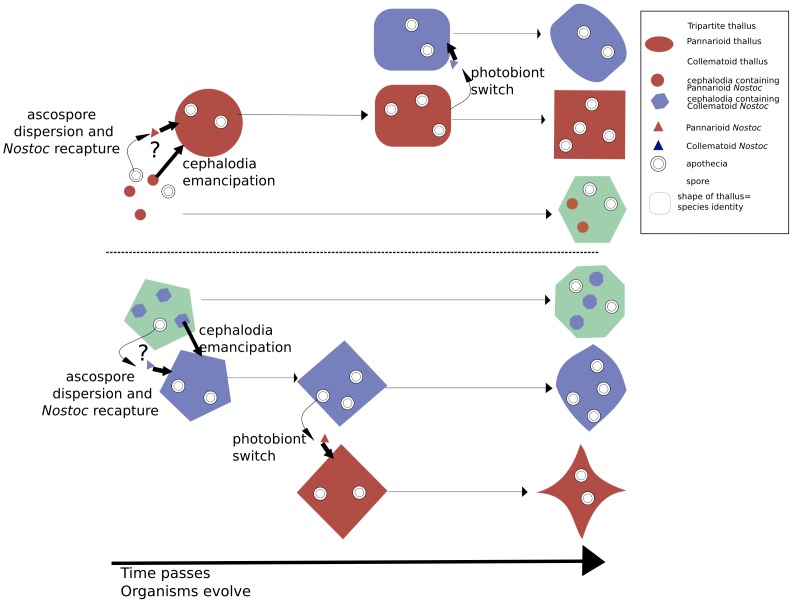
Scheme showing the different scenarios for switching from tripartite to bipartite thallus, and from collematoid to pannarioid thallus and vice versa. Changes in color represent the change of the thallus type. Changes in the shape of the thalli represent the phylogenetic divergence of the different thallus types.

In figure 4, we illustrate the different possible scenarios to switch from tripartite to bipartite, and from collematoid to pannarioid thalli and vice versa, and emphasize on the possibility to obtain, with switches and time, the three types of thalli from the same tripartite ancestor.

As a matter of fact, earlier workers came close to the conclusion that cephalodia can emancipate and start their own evolutionary trajectory. Ekman & Jørgensen [93] pointed to the « homology » between the cephalodia of the green algae-containing *Psoroma hypnorum* and the thallus of the cyanobacterial autonomous species *Santessoniella polychidioides*; Passo et al. [96] retrieved the latter as sister to *Psoroma aphthosum*, a green algal species with coralloid-subfruticose cephalodia, very much akin the thallus of *Santessoniella polychidioides*. We strongly suspect this case represents a further case of cephalodia emancipation, and subsequent divergence. This scenario implies that emancipated cephalodia can reproduce sexually as most species of *Physma* and *Santessoniella polychidioides* produce apothecia and well-developped ascospores. There is indeed no reason to believe that thalli newly formed by cephalodia emancipation and containing only *Nostoc* as photobiont would not be able to produce apothecia, as only the mycobiont is involved in such formation. An interesting alternative would be that, when expelled out of the ascus, the ascospore produced by the mycobiont involved in the ancestral tripartite thallus, would collect or recapture the *Nostoc* of the cephalodia.

Several representatives of the Lobariaceae produce photomorphs, mainly within the genera *Lobaria* and *Sticta* [14], [99]. These duos involving the same fungus lichenized either with a green algae or with a *Nostoc* comprize thalli morphologically rather similar or not (see Introduction), and living attached (thus forming tripartite thalli) or not. Although molecular studies on these duos have mainly sought to demonstrate the strict identity of the fungus involved in each part, the separation or “living apart” of one from the other has long been recognized in several taxa, such as *Lobaria amplissima* and its cyanomorph *Dendriscocaulon umhausense* and *Sticta canariensis* and its cyanomorph *S. dufourii* [100]. There is a priori no reason to exclude that the duos can separate on ”a permanent basis” and thus emancipate; each morph would eventually run its own evolutionnary trajectory, as recently suggested for divergence patterns in *Sticta* photomorphs [101]. Such a scenario can be interpreted as a variant of cephalodia emancipation as advocated here for the evolution of thallus types within the Pannariaceae.

The alternative scenario for the complex phylogenies including bi- and tri-partite thalli implies that a cyanolichen would capture a green algae from the environment (or from another lichen), adopt it as its main photobiont and confine its *Nostoc* into cephalodia. This hypothesis has been suggested by Miadlikowska & Lutzoni [32] for the sect. *Peltidea* in the genus *Peltigera* but so far has not been confirmed. Our data and reconstruction of ancestral state do not support it in the Pannariaceae, with a possible exception for *Joergensenia cephalodina*, but a better sampling is needed in that group to reconstruct the ancestral states.

## Conclusions and Perspectives

Field observations of the lichen species belonging to the widespread and well-known order Peltigerales on the tiny and remote island of Reunion in the Indian Ocean instigated our studies on the relationships between photomorphs in the Lobariaceae (14) and the present study on the Pannariaceae. Indeed, we were intrigued by the occurrence, several times at the same locality or even on the same tree, of representatives of that family with collematoid and pannarioid thalli, and more locally of tripartite thalli.

Collematoid and pannarioid thalli are represented throughout the Pannariaceae. Each thallus type mostly appears mingled within complex topologies. Switches between those thallus types are thus frequent throughout the family. We could demonstrate that both collematoid genera in the Pannariaceae we examined from Reunion material (*Kroswia* and *Physma*) are involved in photobiont switches. We suspect that such a scenario could be detected elsewhere in the Pannariaceae and may act as an important evolutionary driver within the whole family, and perhaps elsewhere within the fungi lineages containing lichenized taxa.

The tripartite thallus type is shown to be the ancestral state in the clade we could study (the *Physma* gr.). Although a larger sampling is needed before such an result could be confirmed, we can postulate that cephalodia emancipation and subsequent evolutionary divergence is the most likely scenario within that clade. The data available support the same scenario in other clades of the Pannariaceae, and it can be suspected in the Lobariaceae where it is represented by the separation and subsequent divergence of photomorphs.

The photomorph pattern in the Lobariaceae demonstrates that a single mycobiont can recognize and recruits phylogenetically unrelated photobiont partners and these associations result in morphologically differentiated thalli. We show here that the use of different lineages of *Nostoc* or the association with only one partner instead of two might lead to the same consequences. Recognition of compatible photobiont cells is carried out by specific lectins produced by the mycobiont, characterized by their ligand binding specificity [102]. *Peltigera* species have served as models in the studies of lectins and their involvment in the recognition of symbiotic partners [103]–[106]. A lectin detects compatible *Nostoc* cells at the initiation of cephalodium formation in *P. aphthosa* and this process is highly specific [107], as further demonstrated by experiment of inoculation of several *Nostoc* strains into the cephalodia of the same species [108]. The biochemical process sustaining the recognition of both partners in two lichen species associated with green algae has been elucidated by Legaz et al. [109] and extended to cyanolichens with collematoid thalli by Vivas et al. [110]. The genes coding for two lectins assumed to be involved in photobiont recognition have recently been identified [111]–[112]. Evaluation of the variation of those genes is of tremendous interest in the context of photobiont switching and cephalodia emancipation as lectins have been shown to be under selection pressure by the symbionts in corals [113]–[114] and a coevolutionary process could thus be highlighted and demonstrated in lichenized fungi. A preliminary study with *Peltigera membranacea* material from Iceland could demonstrate a significant positive selection in LEC-2 but not due to variation in photobiont partner [112].

Further research should thus assemble larger dataset of tripartite taxa within the Pannariaceae and reconstruct their evolutionary history, especially as to the fate of their cephalodia. Numerous methods for detecting genes under positive selection are available [115] and could be applied to the Pannariaceae. Genomics studies of lectins associated with photobiont recognition on tripartite taxa as well as those involved in obvious photobiont switches (pannarioid to collematoid and vice versa) could therefore bring to light a nice model of coevolution [116].

The taxonomical consequences of these results are published in a companion paper, dedicated to new taxa and new combinations.

### Data Accessibility

All newly produced sequences are deposited in GenBank.

All matrices used in the analyses are deposited in Treebase.

## Supporting Information

Figure S1 **Phylogenetic relationships in the family Pannariaceae, based on the best ML tree of the analysis on 3 loci (LSU, mtSSU,** ***RPB1*** **).** Values above branches represent ML bootstrap.
(TIFF)
